# Pituitary Neuroendocrine Tumor or Pituitary Adenoma? Let’s Ask the Epigenome!

**DOI:** 10.1007/s12022-025-09879-8

**Published:** 2025-10-08

**Authors:** Matthias Dottermusch

**Affiliations:** Institute of Neuropathology, University Medical Center Hamburg-Eppendorf, Martinistr. 52, 20246 Hamburg, Germany

**Keywords:** Pituitary, PitNET, Adenoma, Methylation, Epigenome

## Abstract

The introduction of the term pituitary neuroendocrine tumor (PitNET) to replace pituitary adenoma has sparked a versatile debate among experts. The controversy surrounding this nomenclature change includes the question of whether these tumors’ biological identity truly corresponds to neuroendocrine tumors. In this meta-analysis, DNA methylation data were interrogated to clarify whether the old or new nomenclature more accurately reflects the epigenome of these tumors. Publicly available DNA methylation data of 100 NETs, 100 PitNETs/adenomas, and 100 adenomas of various origins and lineages were compiled from 18 different publications. Epigenomic signatures characteristic of NETs and adenomas were defined and compared to those of PitNETs/adenomas. Promoter CpG methylation levels were investigated for hallmarks of cellular differentiation. Comparative DNA methylation analyses demonstrated that all 100 PitNETs/adenomas aligned more closely with NETs than with adenomas. Focusing on promoter-associated CpGs moreover confirmed robust epigenomic features associated with neuroendocrine differentiation in PitNETs/adenomas. These findings indicate that PitNETs/adenomas resemble NETs rather than adenomas on the epigenomic level and support PitNET as the biologically more accurate term. Of note, appropriately addressing the broad spectrum of clinical behaviors in these tumors remains a critical issue in the current pituitary tumor classification framework and nomenclature.

**Supplementary Information:**

The online version contains supplementary material available at 10.1007/s12022-025-09879-8.

## Introduction

Following its establishment in the early twentieth century, the term pituitary adenoma was collectively used to describe a common type of intracranial tumor arising in the anterior pituitary gland [1]. In 2017, the International Pituitary Pathology Club challenged this long-standing terminology [2]. It was argued that, while most of these tumors follow an indolent clinical course, the term adenoma does not adequately capture the potential of some tumors to grow invasively or aggressively. The case was also made from a biological perspective, arguing that these tumors share key gene expression features with neuroendocrine tumors (NETs). In an effort to reflect the broad clinical spectrum and the true biology of these tumors, the term pituitary neuroendocrine tumor (PitNET) was proposed, which was aligned with the goal to adopt these tumors into the larger NET classification framework [3]. The PitNET nomenclature gained traction and was subsequently incorporated into the 2021 WHO classification of central nervous system tumors and the 2022 WHO classification of endocrine and neuroendocrine tumors. While both these WHO classification editions introduced the term PitNET, the term adenoma remained in use, with varying degrees of preference and implications regarding its future role [4, 5].

These nomenclature changes have generated significant controversy. While this controversy is versatile, arguments against the new nomenclature mainly come from the clinical perspective, emphasizing that applying a malignant-sounding label to a predominantly indolent tumor is non-beneficial and risks confusion in clinical management [6–8]. Moreover, critics have questioned whether current evidence justifies the biological classification of pituitary adenomas as NETs [6, 7, 9].

The aim of this study was to approach the latter debate from an epigenomic perspective. DNA methylation signatures reflect cellular origins and lineage identities. Such data have been extensively studied and used to reshape classifications in various tumor entities [10–13]. As of today, epigenomic data of pituitary tumors have mostly been used to explore novel molecular subtypes or correlate specifics of epigenomics, histopathology, and clinical features [14–19]. We have not seen attempts to interrogate DNA methylation data to answer the question which terminology fits better: PitNET or pituitary adenoma?

## Methods

### DNA Methylation Data Acquisition

Raw DNA methylation array data and corresponding sample annotations of the following studies were obtained from public repositories: Bhandari et al. [20] (*n* = 15; GSE233604), Bueno et al. [21] (*n* = 16; GSE179175), Capper et al. [13] (*n* = 30; GSE109381), Chan et al. [22] (*n* = 15; GSE117852), Dos Reis et al. [23] (*n* = 7; GSE97466), Dottermusch et al. [14] (*n* = 15; GSE246645), Fiedler et al. [24] (*n* = 16; GSE129364), Jurmeister et al. [25] (*n* = 9; GSE243075), Kober et al. [26] (*n* = 8; GSE115783), Laddha et al. [27] (*n* = 18; GSE118132), Neou et al. [16] (*n* = 24; E-MTAB-7762), Patte et al. [28] (*n* = 13; GSE229203), Rodríguez-Rodero et al. [29] (*n* = 15; E-MTAB-10906), Salomon et al. [18] (*n* = 7; GSE147548), Silva-Júnior et al. [15] (*n* = 16; GSE207937), Tirosh et al. [30] (*n* = 30; GSE134089), Uro-Coste et al. [31] (*n* = 22; GSE293967), and Werr et al. [32] (*n* = 24; GSE261443). Sample selection and dataset composition were guided by both data availability and the aim of assembling a balanced yet diverse tumor series, representative of various tumor entities. The final dataset included 100 NETs from three common primary sites (lung, pancreas, and small intestine) alongside 100 PitNETs/adenomas of the three major transcription factor-defined lineages (SF1, TPIT, and PIT1) and, finally, 100 adenomas of various origins. The latter comprised both endocrine adenomas (i.e., follicular thyroid and adrenal cortical) and non-endocrine adenomas (i.e., colorectal and pleomorphic salivary gland), with the goal of capturing epigenomic signatures that are shared across benign epithelial tumors of different lineages.

### DNA Methylation Data Preprocessing

R software (v4.3.3) [33] was used to perform computational analyses. Raw methylation array data (idat files) were processed using the minfi R package (v1.48.0) [34] utilizing the preprocessNoob function [35]. Probes on sex chromosomes, probes with a detection *p*-value ≥ 0.01, probes with SNPs at the CpG site, and cross-reactive probes were excluded. When combining different types of arrays, probes that were not represented in both the EPIC and the 450K array were excluded.

### Data Dimension Reduction

Uniform manifold approximation and projection (UMAP) dimension reduction and visualization were performed using the umap R package [36] based on beta values of the top 1000 most variable CpGs. The size of the local neighbourhood was set to 18.

### Epigenomic Signature Correlations

Student’s *t*-tests to detect significantly differentially methylated CpGs were performed using the stats R package (v4.3.3) [33]. The mean beta values of NETs and adenomas of the top significantly differentially methylated CpGs were used to define NET-like and adenoma-like epigenomic signatures. Pearson correlations of PitNET/adenoma mean beta values with both these signatures were calculated. Additionally, Pearson correlations with both these signatures were calculated for each sample. Correlations with the adenoma-like signature were negated to reverse direction. In each sample, both correlations were then converted to Fisher’s z-scores, averaged, and then transformed back for a combined Pearson r. Two-sided *p*-values were calculated from the t-distribution and Bonferroni-adjusted based on the number of samples tested.

### Consensus Clustering

Hierarchical consensus clustering was performed using the ConsensusClusterPlus R package (version: 1.66.0) [37]. Pearson correlation was used as the distance metric, with both inner and outer linkage set to Ward.D2. For cluster stability, the top 1000 most variable CpGs were chosen, 90% of which were subsampled for reclustering. The dendrogram was purposefully partitioned to define two clusters (k = 2).

### Gene set enrichment analysis

Gene set enrichment analysis (GSEA) was performed using the clusterProfiler R package (version: 4.10.0) [38]. For gene sets, cell markers of various cell types from different human tissues were retrieved from the CellMarker 2.0 database [39]. Gene sets were transformed into promoter-associated (TSS1500, TSS200, 5′UTR) CpG sets according to the Champ HumanMethylationEPIC probe annotations [40]. CpGs were ranked by signed − log₁₀(*p*-values) derived from Student’s *t*-tests comparing beta values of PitNETs/adenomas and adenomas, with positive values indicating less methylation in PitNETs/adenomas and negative values indicating less methylation in adenomas. Gene set size ranges were set to 60–6000. GSEA *p*-values were adjusted for multiple comparisons using the Holm-Bonferroni method.

## Results

**Fig. 1:**
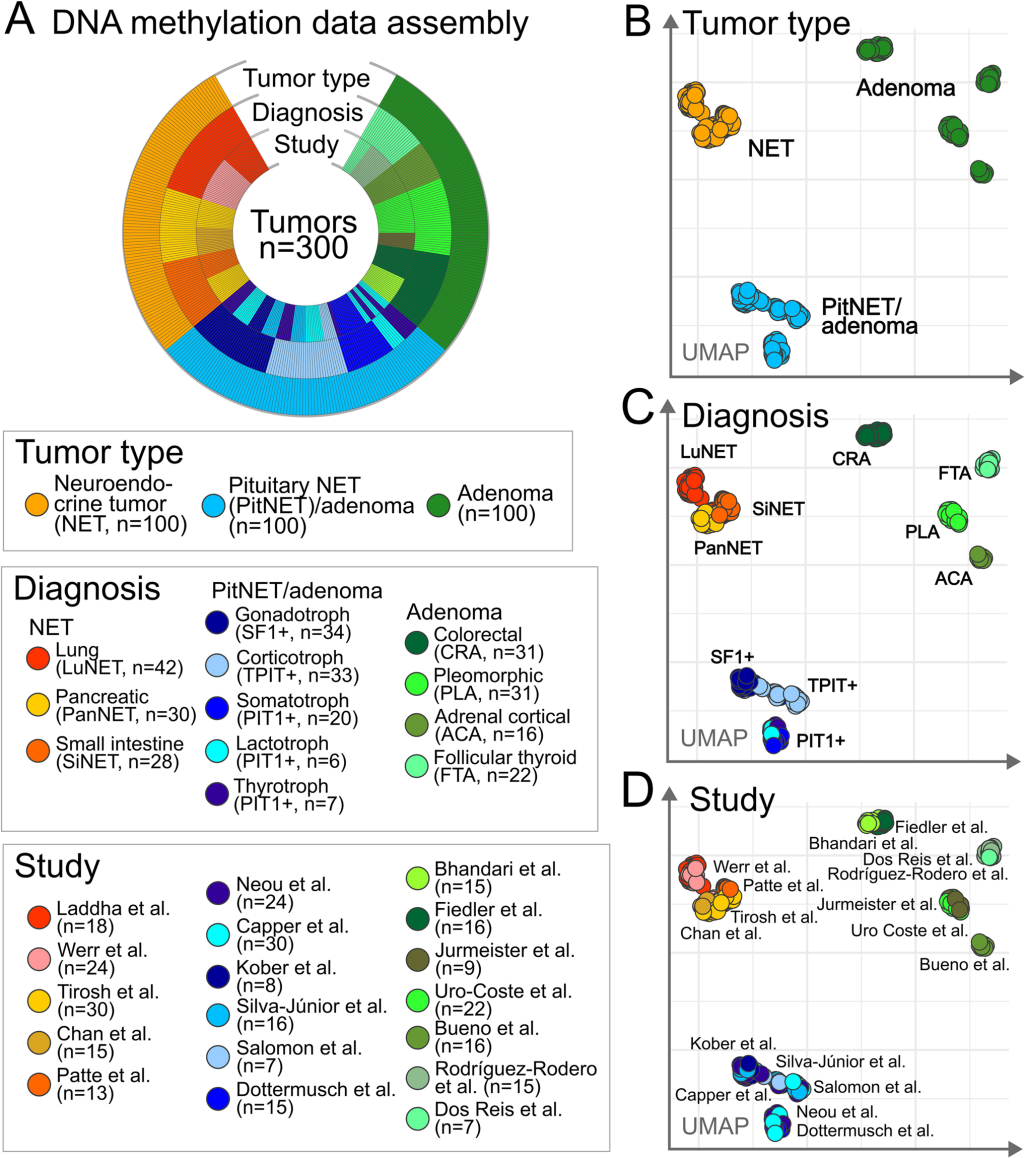
External tumor series assembly and overview. **A** Publicly available global DNA methylation data of a total of 300 tumors (100 NETs, 100 PitNETs/adenomas, and 100 adenomas of various diagnoses) were assembled and integrated from 18 different studies. **B**–**D** Unsupervised dimension reduction shows global epigenomic sample relationships based on beta values. Tumors most notably clustered according to specific diagnoses (**B** + **C**). Batch effects relating to different studies were not apparent (**D**)

Publicly available DNA methylation data of 100 NETs, 100 PitNETs/adenomas, and 100 adenomas of various lineages and tissue origins were compiled from 18 independent previously published studies (Fig. 1A, Supplementary Table 1). As expected, global epigenomic signatures primarily distinguished tumor samples according to their specific diagnoses. In detail, NETs, PitNETs/adenomas, and individual adenoma types formed the most clearly distinguishable epigenomic clusters without apparent batch effects (Fig. 1B–D).

**Fig. 2:**
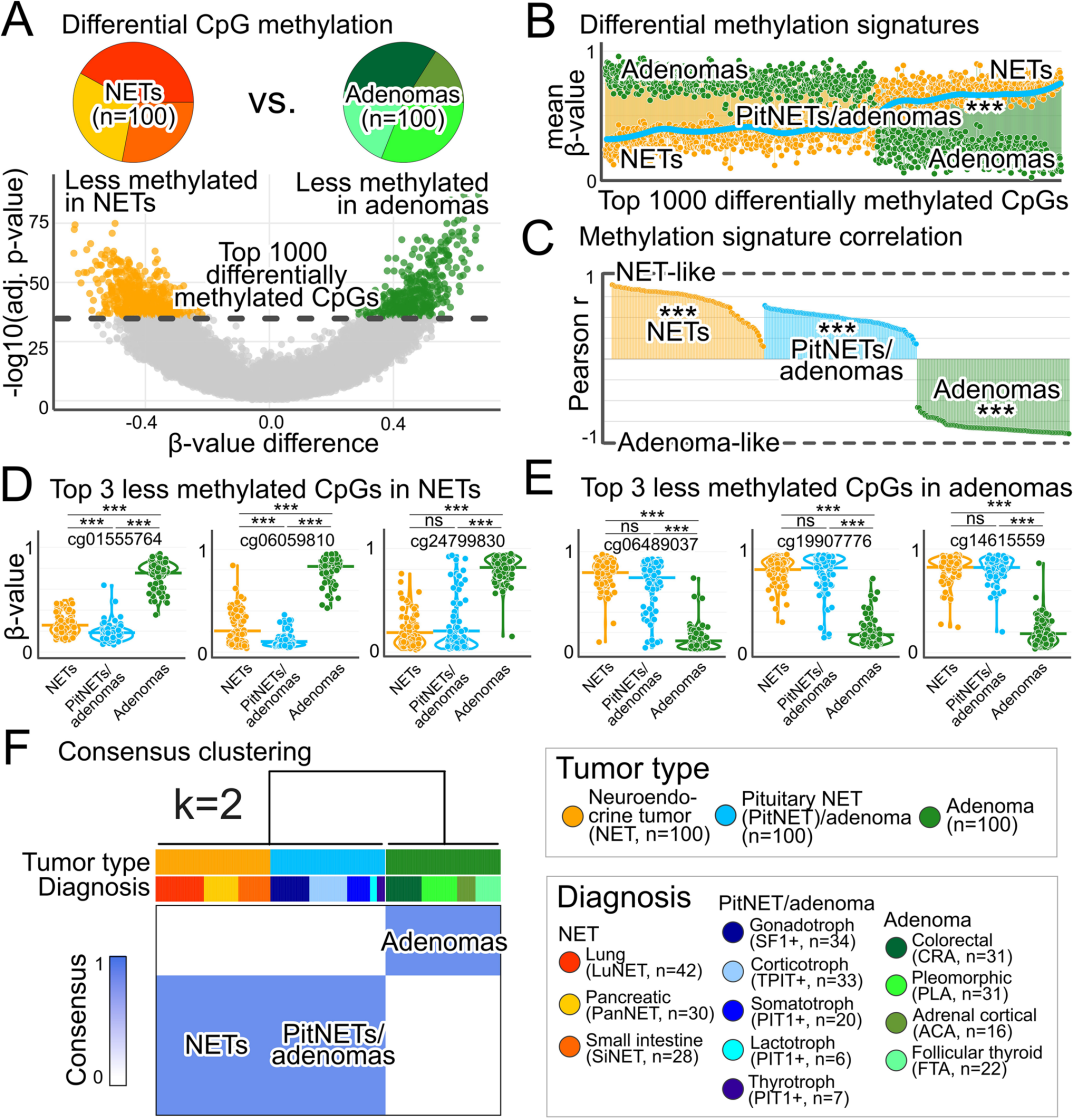
PitNET/adenoma epigenomics align with NETs rather than adenomas. **A** Volcano plot illustrates differential CpG methylation comparing NETs with adenomas. PitNETs/adenomas were excluded from this comparison. Holm-Bonferroni-adjusted *p*-values. **B** The top 1000 significantly differentially methylated CpGs were used to define NET-like and adenoma-like methylation signatures. Data points show the mean beta values of the respective tumor types for each CpG. Colors of the vertical connecting lines correspond to the colors of the CpGs in **A**. The blue line shows a smoothened projection of PitNET/adenoma mean beta values, fitted using local regression with a span of 0.3. PitNET/adenoma beta values aligned more closely with the NET-like signature. Pearson correlation: *r* = 0.74 vs. NETs, *r* =  − 0.5 vs. adenomas; ***p < 0.001. **C** Sample-wise correlation analyses with the NET-like and adenoma-like methylation signatures were performed. All PitNETs/adenomas exhibited NET-like rather than adenoma-like methylation signatures. Bonferroni-adjusted *p*-values were < 0.001 in all PitNETs/adenomas. **D** + **E** Violin plots confirm similar methylation levels in NETs and PitNETs/adenomas for the top 3 CpGs with lower (**D**) and higher (**E**) methylation levels comparing NETs vs. adenomas. Mean values are shown as horizontal bars for each group. ***p < 0.001. ns, not significant. Bonferroni-adjusted *p*-values. **F** Consensus clustering based on the top 1000 differentially methylated CpGs additionally confirmed epigenomic similarity of PitNETs/adenomas with NETs. Hierarchical clustering was performed, and two methylation clusters (k = 2) were defined

Based on differential epigenomics comparing NETs with adenomas, NET-like and adenoma-like methylation signatures were defined (Fig. 2A, Supplementary Fig. 1). PitNETs/adenomas demonstrated a highly significant positive correlation with the NET-like signature (*r* = 0.74, *p* < 0.001) and a negative correlation with the adenoma-like signature (*r* = −0.5, *p* < 0.001; Fig. 2B + C, Supplementary Table 2). Inspecting the most significantly differentially methylated CpGs showcased congruent methylation statuses in NETs and PitNETs/adenomas (Fig. 2D + E). Additionally, consensus clustering based on differential epigenomics of NETs and adenomas confirmed the affiliation of NETs and PitNETs/adenomas (Fig. 2F).

**Fig. 3:**
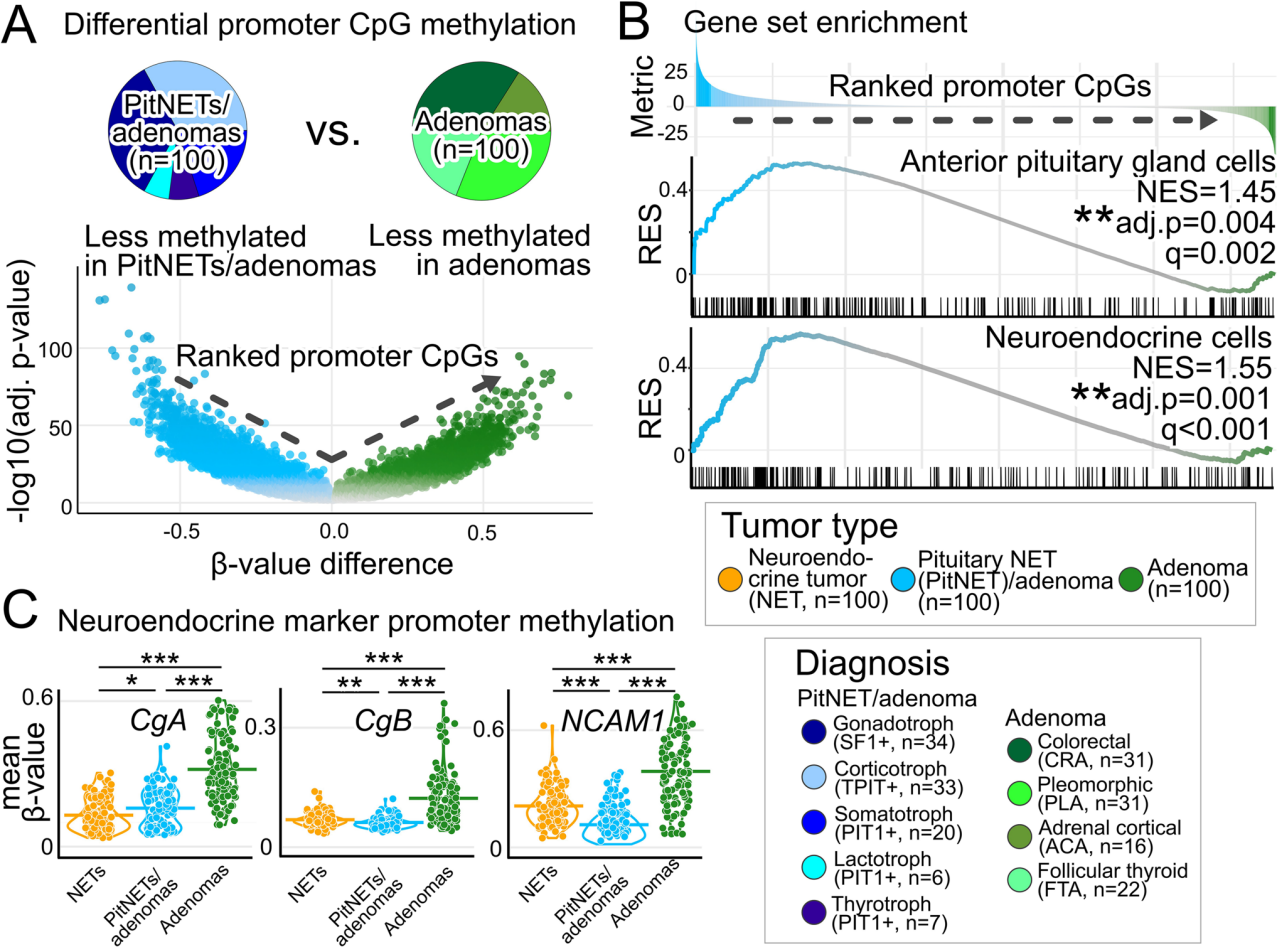
PitNET/adenoma epigenomics reflect neuroendocrine signatures. **A** Volcano plot illustrates differentially methylated promoter-associated CpGs comparing PitNETs/adenomas with adenomas. Holm-Bonferroni-adjusted *p*-values. **B** Promoter-associated CpGs were ranked according to their signed *p*-values (in **A**) for gene set enrichment analyses (GSEA). Notably, cellular signatures “anterior pituitary gland cells” and “neuroendocrine cells” were both highly significantly and positively enriched in PitNETs/adenomas compared to adenomas. RES, running enrichment score; NES, normalized enrichment score. Holm-Bonferroni-adjusted *p*-values. **C** Violin plots confirm significantly less promoter region methylation in NETs and PitNETs/adenomas compared to adenomas for chromogranin A (*CgA*), chromogranin B (*CgB*), and CD56 (*NCAM1*). Mean values are shown as horizontal bars for each group. *p < 0.05. **p < 0.01. ***p < 0.001. Bonferroni-adjusted *p*-values

To answer the question which hallmarks of cellular differentiation were reflected by the epigenome of PitNETs/adenomas, the focus was shifted towards promoter-associated CpGs. Differential epigenomics comparing PitNETs/adenomas with adenomas (Fig. 3A) provided the basis for gene set enrichment analyses (GSEA), which confirmed positive enrichment for “anterior pituitary gland cells” (NES = 1.45, *p* = 0.004) in PitNETs/adenomas, as expected. Moreover, “neuroendocrine cells” (NES = 1.55, *p* = 0.001) and further neuronal-like signatures (Supplementary Table 3) were positively enriched in PitNETs/adenomas (Fig. 3B). Focusing on the promoter methylation status of the neuroendocrine markers chromogranin A (*CgA*), chromogranin B (*CgB*) and CD56 (*NCAM1*) confirmed congruently lower methylation levels in NETs and PitNETs/adenomas compared to adenomas (Fig. 3C).

## Discussion

In this study, multiple publicly available DNA methylation datasets were integrated to answer the question of whether the PitNET/adenoma epigenome is more reflective of NET or adenoma terminology. This meta-analytical approach has some technical limitations. Batch correction implementation was not straightforward, due to the unbalanced batch compositions and the lack of consistent tumor entity representations across them [41]. Complete data integration also necessitated a reduction to 450K array probes, which narrowed the global epigenomic perspective. Nevertheless, as far as assessable, study-related batch effects were non-apparent after applying suitable techniques for integrating different methylation array types [35].

Using various analytical approaches, the results of this study indicate that the epigenomic signatures of PitNET/adenomas are more NET-like than adenoma-like. It is worth noting that global epigenomic profiles were primarily reflective of histopathological tumor diagnoses in this study, as was expected and supported by the literature [13, 42, 43]. Accordingly, PitNETs/adenomas harbored distinct methylation signatures, which made them clearly distinguishable from other tumor entities including non-pituitary NETs. When visualizing highly dimensional data via UMAP plots, it is important to keep in mind that intercluster distances do not directly translate to meaningful degrees of similarity or distinctness [44]. Thus, the distinguished epigenomic profiles of PitNET/adenomas do not contradict the finding that these tumors’ signatures still aligned closer with non-pituitary NETs than with non-pituitary adenomas.

Epigenomic profiles were consistent with neuroendocrine expression features in PitNETs/adenomas. Critics have previously questioned whether neuroendocrine marker expression truly establishes these tumors’ biological classification as NETs [6, 7, 9]. These concerns were partly based on previous immunohistochemical studies reporting neuroendocrine marker expression in non-NET entities [45–48]. Notably, the neuroendocrine immunostainings encountered in these previous studies’ tumors were often limited and rarely described as strong. The epigenomic analyses of this study support the neuroendocrine identity of PitNETs/adenomas, reinforcing conclusions previously drawn from immunohistochemistry [49, 50].

It should be noted that the herein presented findings do not alter the fact that the majority of PitNETs/adenomas follow an indolent course and are clinically considered benign. The parts of the controversy surrounding the renaming of pituitary adenomas to PitNETs, which are rooted in the malignant implications of the current PitNET classification framework via the /3 ICD-O code, remain unaddressed in this study. Future studies working towards establishing reliable prognostic markers in PitNETs/adenomas may help resolve this issue.

## Conclusion

DNA methylation analyses confirm epigenomic signatures aligned with NETs in PitNETs/adenomas. The epigenomic perspective speaks in favor of PitNET being the biologically more accurate term. How to solve the issue of appropriately addressing the broad spectrum of clinical behaviors in PitNETs/adenomas remains a critical question for the current PitNET classification framework.

## Supplementary Information

Below is the link to the electronic supplementary material.

ESM 1 (XLSX 22.4 KB)

ESM 2 (XLSX 29.8 KB)

ESM 3 (XLSX 85.4 KB)

ESM 4 (JPEG 5.54 MB)

## Data Availability

The datasets processed in this study were previously published and are publicly accessible via their original sources cited in this work.
